# Role of Dietary Macronutrients and Fatty Acids in Obesity and Metabolic Risk in Older Adults

**DOI:** 10.18689/ijons-1000102

**Published:** 2019-07-19

**Authors:** Cara Dooley, Alice S Ryan

**Affiliations:** 1 University of Maryland School of Medicine, Baltimore, USA; 2 VA Research Service, VA Maryland Health Care System, Baltimore, USA; 3 Department of Medicine, Division of Gerontology and Geriatric Medicine, University of Maryland School of Medicine, Baltimore, USA; 4 Baltimore VA Medical Center Geriatric Research, Education and Clinical Center (GRECC), Baltimore, USA

**Keywords:** Hyperlipidemia, Macronutrients, Fatty acids, Diabetes, Obesity

## Abstract

The aim of the study was to examine the role of dietary consumption of different types of fatty acids on metabolic risk factors and regional fat deposition in older men and women. We hypothesized that saturated fatty acid (SFA) intake, monounsaturated fatty acid (MUFA) and low intake of polyunsaturated fatty acids (PUFA) would be associated with markers of insulin resistance, hyperlipidemia, and hypertriglyceridemia. Sedentary, overweight and obese (body mass index: 29–48 kg/m^2^) adults (N=20) aged 45–78 years underwent two-hour oral glucose tolerance test, blood draw, DXA scan, and completed seven-day diet records. Subjects had low fitness levels (VO_2_ max=23.5 ± 2.4 mL/kg/min) and high total body fat (43.5 ± 1.7%). The average macronutrient composition of the diet was high in fat as a percent of total kcal (35.5%). The ratio of MUFA to PUFA was associated with serum cholesterol (r=0.48, P=0.03) and tended to be associated with higher fasting glucose (r=0.42, P=0.06) and glucose at 120 min (r=0.43, P=0.06). PUFA intake as a percentage of fat intake was associated with lower serum cholesterol (r=−0.44, P=0.05). Therefore, dietary MUFA intake unbalanced by PUFA may confer increased risk for diabetes among obese, sedentary individuals. Future investigation of food sources, or context of dietary lipids, could lead to individualized dietary recommendations to promote healthy eating habits and potentially alter metabolic risk.

## Introduction

Dietary fat composition is highly modifiable, and possibly one of the most important factors influencing insulin resistance and blood cholesterol. In the last several decades, unsaturated fatty acids have gained importance as a dietary component that could prevent chronic disease, with saturated fats generally regarded as detrimental to one’s health [1]. Dietary fatty acids determine plasma availability and tissue storage of lipids, and have long been speculated to exert an effect through altering cell membrane composition and function particularly in skeletal muscle, as well as gene expression, enzyme activity, and immune cell function [2–6].

Several large epidemiological studies support the association between dietary saturated fat intake and type two diabetes risk [7–9]. In a large prospective study, development of diabetes over nine years was associated with saturated fatty acid (SFA) in serum cholesterol and phospholipids with adjustment for diabetes risk factors [10]. A large prospective case-cohort study of Pima Indians demonstrated an association between dietary fatty acids, serum saturated fatty acids and self-reported diabetes four years later [11]. Dietary linoleic acid (LA), a polyunsaturated fatty acids (PUFA) estimated to account for 85–90% of dietary PUFA, was inversely associated with diabetes risk [11,12]. Likewise, in the prospective Nurses’ Health Study, polyunsaturated fat intake was reported to lower relative risk of diabetes [13].

Obese adults and those with type-two diabetes are known to have higher circulating levels of free fatty acids than non-obese non-diabetic individuals [14]. Cross-sectional studies demonstrated that lean individuals have a greater proportion of LA, in serum fatty acids than obese individuals, who tend to have more serum fatty acid in general, and a greater proportion of saturated fatty acid intake such as palmitate [15]. MUFA and PUFA are thought to benefit serum lipid profiles by limiting hepatic steatosis and subsequently increasing lipid metabolism and decreasing lipogenesis [3,16]. A controlled study of supplemented LA intake demonstrated favorable decreases in total and LDL cholesterol but HDL and triglycerides were unchanged [17]. High PUFA diet has been shown to decrease LDL in small controlled studies, and high SFA to increase LDL [16,18]. Yet, studies analyzing MUFA in relation to PUFA on diabetes risk and its effect on serum lipid profile are limited in number [16].

The aim of the study was to examine the role of dietary consumption of different types of fatty acids on metabolic risk factors in middle-aged and older men and women. We hypothesized that SFA intake, and low intake of MUFA and PUFA would be associated with markers of insulin resistance, hyperlipidemia, and hypercholesterolemia in middle-aged and older overweight and obese men and women.

## Methods

Male and female adults aged 45–80 years from the Baltimore/Washington area were eligible if overweight or obese (BMI 29–50 kg/m^2^) but otherwise healthy. Women were included only if they had undergone menopause for at least one year. Only adults who were weight stable (<2.0 kg weight change in past year) and sedentary (<20 min of aerobic exercise 2x/week) were recruited. Men and women were excluded for the presence of heart disease, diabetes, cancer, anemia, dementia untreated dyslipidemia, as well as those with other unstable or chronic diseases affecting the liver, lungs, or kidneys. Potential participants were screened and underwent a physical examination including a comprehensive past medical history, fasting blood profile, and a graded exercise treadmill test. Each study participant provided written consent. All methods and procedures were approved by the Institutional Review Board at University of Maryland and the VA Research and Development Committee.

### Nutrition

Subjects were instructed by a registered dietician (RD) on how to complete a food record. Each subject completed a 5-day food record and recorded all foods eaten, and the estimated quantity of each food. Diet records were analyzed for macronutrient content, with lipids further recorded as SFA, MUFA, PUFA, omega 3, omega 6, Eicosapentaenoic Acid (EPA), Docosahexaenoic acid (DHA), trans-fatty acid and cholesterol, as well as sodium and fiber using Nutritionist Pro software. Additional analysis of the food records were performed with the USDA Foodapedia feature of the Super tracker program.

### VO_2_ max

VO_2_max was measured using a continuous treadmill test protocol [19].

### Body composition

Height (cm) and weight (kg) were measured to calculate BMI. Percent body fat mass was determined by dual-energy X-ray absorptiometry (iDXA, LUNAR Radiation Corp, Madison, WI).

### Oral Glucose Tolerance Test (OGTT)

After a 12-hour overnight fast, the subjects had a blood draw before and at 30-min intervals for 2 h after ingestion of 75 g glucose. Samples were collected in heparinized syringes, placed in pre chilled test tubes containing 1.5 mg EDTA/ml blood, centrifuged at 4°C and stored at −80°C until analysis. Plasma glucose concentrations were measured using the glucose oxidase method (2300 STAT Plus, YSI, Yellow Springs, OH). Plasma insulin was measured in duplicate by radioimmunoassay (RIA) (Millipore, St. Charles, MO). The subjects were defined by glucose tolerance status.

### Lipoprotein lipids

Plasma triglyceride and cholesterol levels were averaged from two fasting blood draws analyzed using enzymatic methods (UniCel DxC880i, Beckman Coulter, Inc., Brea, CA) and high-density lipoprotein cholesterol (HDL-C) measured in the supernatant after precipitation with dextran sulfate (low-density lipoprotein cholesterol (LDL-C)=total cholesterol–(TG/5 + HDL-C)) [20].

### Statistical analyses

Descriptive means were calculated on variables. Unpaired t-tests were used to test differences by sex. Pearson correlations and partial correlations were used to assess relationships between key variables. Statistical significance was set at a two-tailed P<0.05. Data were analyzed using SPSS (SPSS Inc., Chicago). Results are expressed as mean ± SEM.

## Results

### Phenotype and diet composition

Descriptive characteristics of the subjects are provided in table 1. The subjects were 70% Caucasian and 30% African American and included 9 men and 11 women. Subjects had low fitness levels (VO_2_ max: 23.5 ± 2.4 ml/kg/min) and high total body fat (43.5 ± 1.7%). Fasting and OGTT results as well as lipoprotein lipid results are shown in Table 1. Twelve subjects had normal glucose tolerance (NGT) and seven subjects had impaired glucose tolerance (IGT).

Dietary characteristics of the subjects are compared to national dietary reference intakes (DRI), including accepted macronutrient distribution range (AMDR) or adequate intake (AI), and are presented in Table 2 [21]. The average macronutrient composition of the diet is fairly homogenous. Diets were high in proportion of fat with roughly equal percentages of SFA and MUFA, whereas intake of PUFA intake had the lowest percent of total fat intake. No significant differences were noted in the diets between NGT and IGT subjects for percent of calorie intake for carbohydrate, protein, and fat (data not shown) or for daily intake of MUFA (31.0 ± 2.7 vs. 28.7 ± 3.4 g), PUFA (20.3 ± 1.9 vs. 18.2 ± 2.7 g) and SFA (29.3 ± 2.5 vs 27.0 ± 3.4 g), respectively.

Men on average consumed more total calories per day than women (2506 ± 110 vs. 1948 ± 110 kcal/d, P<0.005). Although men had higher carbohydrate, protein, and fat intake than women (all P<0.05), there were no significant differences between men and women for percent calories from carbohydrate (47.1 ± 1.9 vs. 49.0 ± 2.0), percent calories from protein (15.9 ± 0.8 vs. 16.5 ± 0.5), and percent calories from fat (35.8 ± 0.7 vs. 35.2 ± 1.6). Men had higher total MUFA intake than women (36.3 ± 2.0 vs. 25.8 ± 2.4 g, P<0.01) and MUFA as a percent of total fat intake (36.4 ± 1.0 vs. 33.4 ± 1.1%, P=0.05), with no difference between men and women in PUFA intake (22.0 ± 0.7 vs. 17.5 ± 2.5 g) or SFA intake (32.6 ± 3.2 vs. 25.5 ± 1.9 g).

### Relationships of diet with glucose tolerance and lipids

The ratio of total MUFA to PUFA was associated with serum cholesterol and tended to be associated with markers of glucose intolerance including fasting glucose and glucose AUC (Table 3). Total MUFA to PUFA ratio was not associated with fasting insulin or insulin AUC. Average daily PUFA intake tended to be associated with lower glucose at 120 min of the OGTT (r=−0.43, P=0.08). PUFA intake as a percentage of fat intake and the ratio of PUFA:SFA was associated with lower serum cholesterol (r=−0.44, P=0.05 and r=−0.40, P=0.07, respectively). Additionally, the ratio of omega-3 to omega-6 fatty acids was negatively correlated to insulin AUC (r=−.054, P=0.01).

## Discussion

Our results indicate that overweight and obese subjects have a high dietary intake of fat as a percent of total caloric intake, especially SFA and MUFA. The dietary intake of SFA, MUFA and PUFA in our sample closely resembles those of the average American using NHANES data [22]. We did not find differences in dietary intake of fatty acids between adults with normal and impaired glucose tolerance. Greater MUFA intake compared to PUFA tended to be associated with glucose intolerance and higher serum cholesterol in these overweight and obese sedentary subjects.

Our results would suggest that dietary intake of PUFA confer some reduction in metabolic risk in terms of glucose metabolism and hypercholesterolemia. In a systematic review and meta-analysis of randomized controlled feeding trials, replacing carbohydrate intake with PUFA or replacing SFA with PUFA reduces blood glucose levels and insulin resistance and improves insulin secretion [23]. The association between PUFA intake and lower total cholesterol further support a potentially beneficial effect of PUFAs on various aspects of lipid metabolism.

In a small meta-analysis of four studies of adults with type-two diabetes that compared high-MUFA to high-PUFA diets, the MUFA groups had reductions in fasting plasma glucose [24]. Although our results appear to contradict this analysis, MUFA intake is confounded by its food source as it can reflect either consumption of meat and dairy products containing oleic acid, or of non-hydrogenated vegetable oils like olive oil or avocado. Thus, detailed food records of intake are necessary to further elucidate any conclusions.

The proportion of omega-3 intake to omega-6 may partly explain greater insulin sensitivity. This would be in concert with epidemiologic studies drawing attention to increasing ratios of omega-6 to omega-3 in the American diet over time and associations with obesity, as well as benefits to insulin sensitivity with supplementation of omega-3 fatty acids [25,26].

We did not find any significant associations of saturated fat intake with metabolic risk, likely due to the homogenous sample of overweight and obese adults. Our findings align with the longitudinal findings of the Nurses’ Health Study, where Type 2 diabetes risk was not associated with total fat intake, saturated or monounsaturated fat intake [13]. Furthermore, analysis of a single fats alone, or proportions of fat groups in a dietary pattern, may not have been sufficient to relate to metabolic outcomes. In fact, inverse association are reported with percentage of energy derived from fat, SFA, MUFAs and PUFAs suggesting that overall composition may play a more important role than total amount of each type of fatty acid [27]. Using the National Health and Nutrition Examination Survey (NHANES) of over 24,000 participants, Mazidi et al. [28], reported that subjects who adhered to dietary patterns determined by a one-day 24 h food record either comprised of SFA, total fat, MUFA and CHO and or composed of PUFA, cholesterol, and protein had a higher likelihood of developing insulin resistance.

Analysis of diet composition in terms of individual nutrients should put the food in context; specifically that food is not composed simply of individual nutrients, but is a complex interplay of micro and macronutrients, influenced by its source, preparation, and by the other foods in a meal. This multi-layered context of a nutrient, as well as the metabolic characteristics of an individual, will determine the uptake, use, storage of said nutrient, as well as long term metabolic sequelae of any diet patterns.

Limitations of this study include the small sample size, relatively homogenous nature of the group, and inability to control for potential confounding factors such as age, sex, and race. In addition, the cross-sectional design of the present study does not allow investigation of the impact of dietary fat composition on the development of diabetes as in a follow-up study. However, the strengths of the study include a thorough analysis of dietary intake with a 5-day dietary intake and careful characterization of the subjects including measures of fitness by VO_2_ max testing, glucose metabolism by oral glucose tolerance test, and lipoprotein lipid profiles which were measured twice and averaged.

## Conclusion

In conclusion, dietary MUFA intake, unbalanced by PUFA was associated with glucose intolerance and increased serum cholesterol, and therefore may confer increased risk for diabetes among obese, sedentary individuals. Further investigation could potentially aim to analyze the source of dietary fat, as well as meal patterns. Individualized dietary recommendations could therefore aim to incorporate food source and context of dietary lipids to promote healthy eating habits and potentially alter metabolic risk.

## Figures and Tables

**Table 1.** Subject Characteristics.

Variables	N=20
Age (yrs)	59 ± 2
Weight (kg)	98.6 ± 1.8
BMI (kg/m^2^)	34.8 ± 1.0
Waist Circumference (cm)	107.4 ± 3.3 (n = 18)
Fasting Glucose (mg/dL)	98 ± 3
2 hr glucose OGTT(mg/dL)	139 ± 9
Glucose AUC (mg/dL120 min)	18204 ± 878
Fasting Insulin (pmol/L)	116 ± 12
2 hr Insulin OGTT (pmol/L)	681 ± 74
Insulin AUC (pmol/L.120 min)	77543 ± 8640
Triglycerides (mg/dL)	125 ± 13
Low-density lipoprotein (mg/dL)	110 ± 5
High density lipoprotein (mg/dL)	46 ± 3
Cholesterol (mg/dL)	181 ± 6

**Table 2.** Dietary Characteristics.

Variables	Mean ± SEM (N=20)	AMDR, AI, UL
Caloric intake (kcal/day avg)	2200 ± 99	n/a
%Kcal from Fat	35.5 ± 0.9	20–35
%Kcal from CHO	48.1 ± 1.3	45–65
%Kcal from Protein	16.2 ± 0.4	10–35
MUFA % Total Fat	34.8 ± 0.8	n/a
PUFA % Total Fat	22.1 ± 1.2	n/a
SFA % Total Fat	33.0 ± 1.1	n/a
MUFA %Total kCal	12.3 ± 0.4	n/a
PUFA % Total kCal	8.0 ± 0.5	n/a
SFA % Total kCal	11.7 ± 1.2	n/a
MUFA:PUFA Ratio	1.66 ± 0.1	n/a
PUFA:SFA Ratio	0.7 ± 0.1	n/a
Omega-3 fatty acid (mg/day)	1.9 ± 0.2	0.6–1.2
EPA (g/day)	0.11 ± 0.1	n/a
DHA (g/day)	0.12 ± 0.2	n/a
Omega-6 fatty acid (mg/day)	16.2 ± 1.2	5–10
Omega-3: Omega-6 ratio	0.1 ± 0.01	n/a
Trans Fatty Acid (g/day)	0.7 ± 0.1	n/a
Cholesterol (mg/day)	314.2 ± 26.1	<300
Sodium (mg/day)	3458 ± 210	<2300
Fiber (g/day)	22.9 ± 1.7	30 (male), 21 (female)

**Table 3.** Pearson correlations.

Dietary Variable	Metabolic Variable	Pearson Correlation (r)	P-value
MUFA:PUFA	Fasting glucose	0.42	0.06
	Glucose at 120 min	0.42	0.06
	Glucose AUC	0.41	0.07
	Fasting insulin	−0.18	0.45
	Insulin AUC	−0.22	0.36
	Cholesterol	0.48	0.03
	Triglycerides	0.33	0.16
	LDL-cholesterol	0.38	0.09

## Abbreviations:

AEX: Aerobic Exercise
BMI: Body Mass Index
OGTT: Oral Glucose Tolerance Test
AUC: Area Under Curve
TLC: Therapeutic Lifestyle Changes
MUFA: Monounsaturated Fatty Acid
PUFA: Polyunsaturated Fatty Acid
SFA: Saturated Fatty Acid
LA: Linoleic Acid
EPA: Eicosapentaenoic Acid
DHA: Docosahexaenoic acid
DRI: Dietary Reference Intake
AMDR: Accepted Macronutrient Distribution Range
AI: Adequate Intake
UL: Upper Limit
